# Successful Management of Severe Hypertriglyceridemia Presenting With Eruptive Xanthomas as an Outpatient Without Development of Acute Pancreatitis: A Case Report

**DOI:** 10.7759/cureus.110536

**Published:** 2026-06-09

**Authors:** Rinsila R Hafthar, Rahma A Alibare, Madihah Abdul Afeez, Abdul Aziz

**Affiliations:** 1 Internal Medicine, Leicester Royal Infirmary, University Hospitals of Leicester NHS Trust, Leicester, GBR; 2 Acute Medicine, Leicester Royal Infirmary, University Hospitals of Leicester NHS Trust, Leicester, GBR

**Keywords:** acute pancreatitis (ap), diabetes mellitus, eruptive xanthoma, familial hypercholestrolemia, hypertriglyceridemia (htg), hypertriglyceridemia-induced acute pancreatitis, severe hypertriglyceridemia

## Abstract

Eruptive xanthomas are a rare manifestation of severe hypertriglyceridemia and are commonly associated with uncontrolled diabetes mellitus and an increased risk of acute pancreatitis. We report the case of a 41-year-old man who presented with diffuse papular skin lesions and neuropathic symptoms. He had no previous diagnosis of diabetes; however, initial investigations revealed a markedly elevated glycated hemoglobin (HbA1c) of 13.8%, and he was subsequently diagnosed with diabetes mellitus. Further testing showed severe hypertriglyceridemia (85.6 mmol/L), elevated cholesterol, and widespread eruptive xanthomas on examination. Despite the markedly raised triglyceride level, he remained clinically stable and reported no abdominal pain, nausea, or vomiting, with no abdominal tenderness on examination to suggest acute pancreatitis. He was managed as an outpatient with insulin therapy, a statin, a fibrate, and lifestyle modification. This led to a rapid improvement in triglyceride levels, glycemic control, and cutaneous lesions, with no development of pancreatitis. This case highlights eruptive xanthomas as a potential initial presenting feature of previously undiagnosed diabetes mellitus with severe hypertriglyceridemia, and illustrates that carefully selected clinically stable patients may be managed in the outpatient setting with early recognition and prompt metabolic optimisation.

## Introduction

Extremely elevated triglyceride levels can cause lipids to accumulate in the skin causing eruptive xanthomas [1]. Eruptive xanthoma is a rare dermatologic manifestation of severe hypertriglyceridemia and chylomicronemia [2]. It is strongly associated with metabolic syndrome [3]. Eruptive xanthomas are caused by severe hypertriglyceridemia (triglycerides >11.2 mmol/L) [2].

Eruptive xanthomas have an estimated prevalence of 18 cases per 100,000 people and have been documented retrospectively in 10% of patients with severe hypertriglyceridemia [4,5]. In patients with severe hypertriglyceridaemia (triglycerides >20 mmol/L), the prevalence of eruptive xanthomas was 8.5% [2]. It is inherited in an autosomal recessive manner [2]. It can also occur secondary to diabetes mellitus [6]. Hypertriglyceridemia is linked to a higher risk of cardiovascular events and acute pancreatitis [7].

## Case presentation

A 41-year-old man presented with a four-to-six-month history of chronic tingling and numbness in both lower limbs. He had been investigated in the community, where significantly elevated triglyceride levels were identified on biochemistry. The results of the initial blood tests, including triglycerides, cholesterol, and glycated hemoglobin (HbA1c), are shown in Table 1.

**Table 1:** Initial laboratory investigations on presentation HbA1c: glycated hemoglobin

Investigation	Patient value	Reference range
Triglycerides	85.62 mmol/L	0-2 mmol/L
Total cholesterol	21.6 mmol/L	<5 mmol/L
HbA1c	13.8%	4.0-5.9%

He was therefore referred to the Medical Same Day Emergency Clinic (M-SDEC) for further assessment, and the investigations performed on the following day are summarised in Table 2. 

**Table 2:** Repeat laboratory investigations following assessment in the Medical Same Day Emergency Clinic (M-SDEC) HbA1c: glycated hemoglobin; ALT: alanine aminotransferase; eGFR: estimated glomerular filtration rate

Investigations	Patient value	Reference range
Triglycerides	60.57 mmol/L	0-2 mmol/L
Total cholesterol	21.3 mmol/L	<5 mmol/L
HbA1c	13.2%	4.0-5.9%
ALT	<9 IU/L	2-53 IU/L
Total bilirubin	2 umol/L	0-21 umol/L
Alkaline phosphatase	104 IU/L	30 - 130 IU/L
eGFR	>90	>90

The patient also reported diffuse skin lesions affecting the trunk and both upper limbs, which had been gradually progressing over the preceding year. In addition, he complained of excessive sweating and fatigue. He noted a weight gain of approximately 4 stones over the previous four years. His past medical history was significant for anxiety-depressive disorder, for which he was taking venlafaxine. He worked as a DJ (disc jockey) and reported increased alcohol intake. 

**Figure 1:**
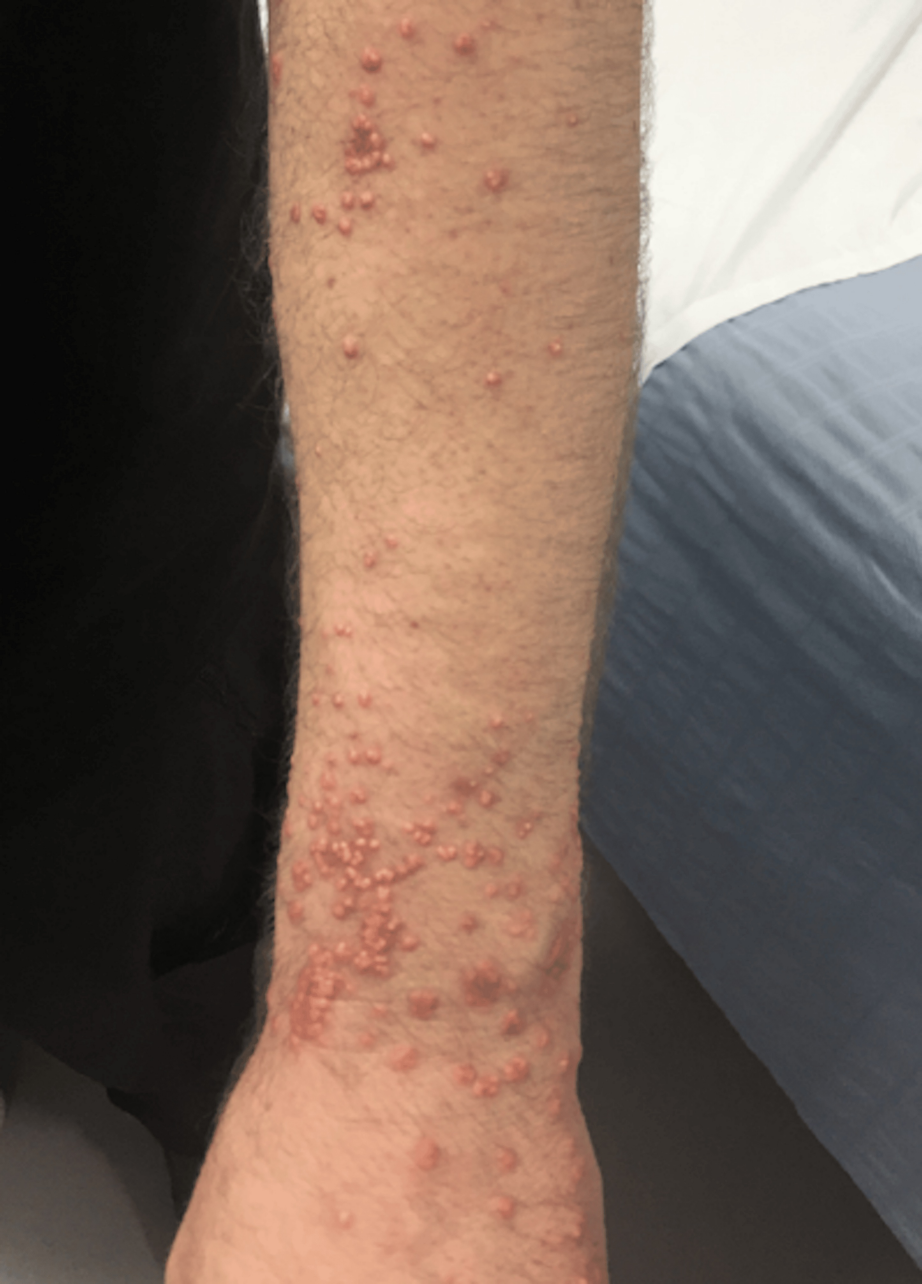
Diffuse papular eruptive xanthomas over the upper limb at presentation.

**Figure 2:**
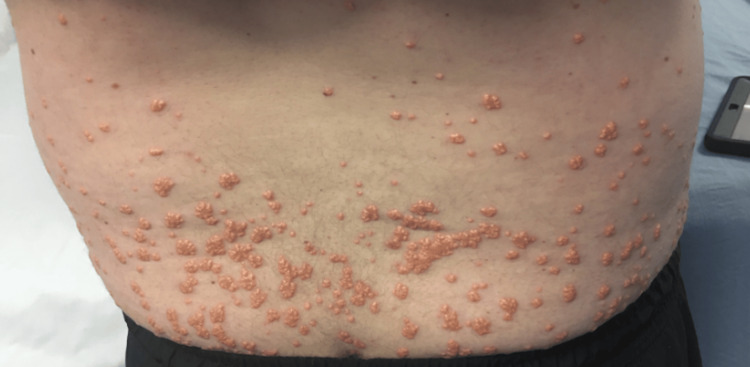
Diffuse papular eruptive xanthomas over the trunk at presentation.

**Figure 3:**
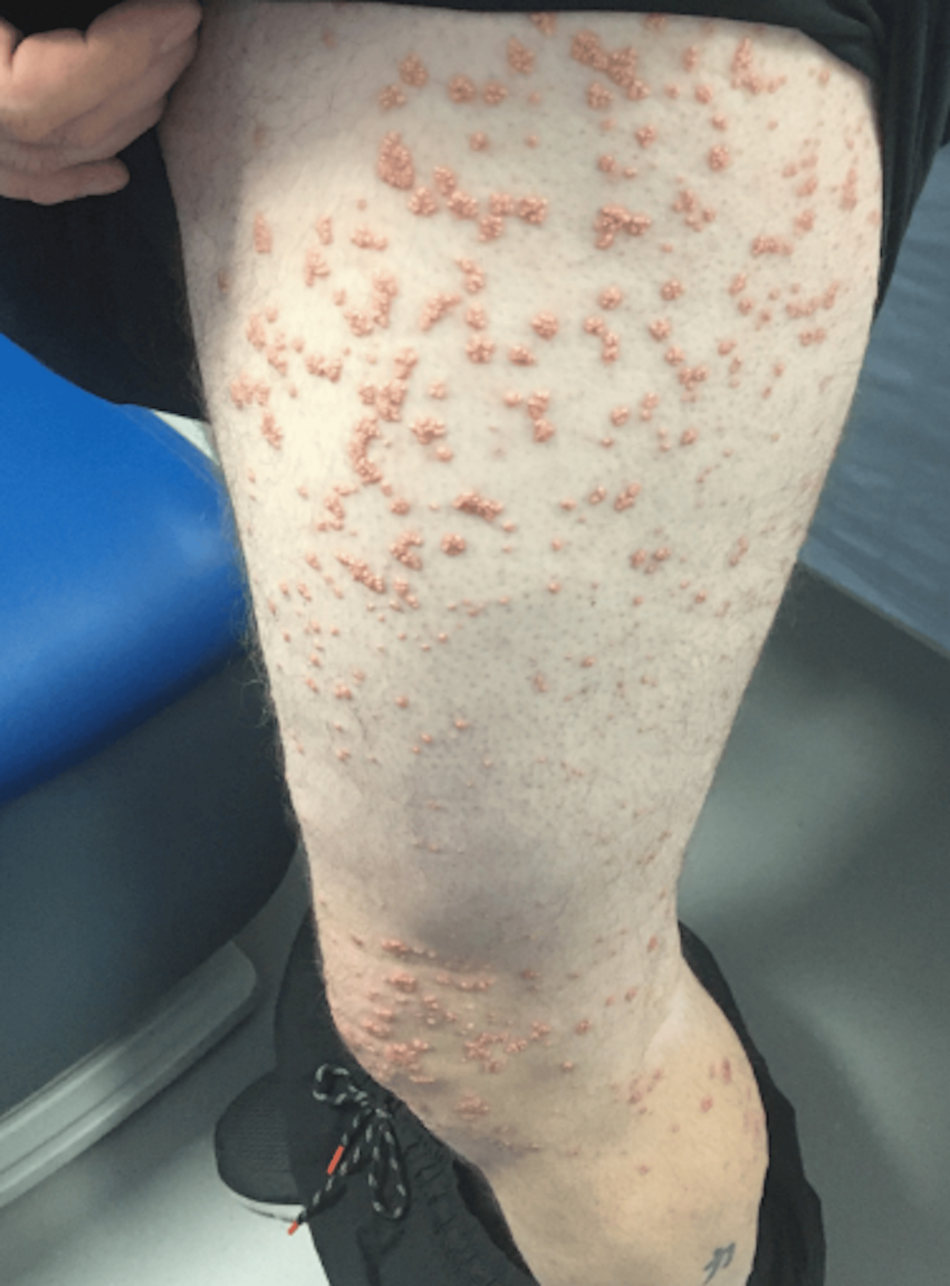
Diffuse papular eruptive xanthomas over the lower limb at presentation.

**Figure 4:**
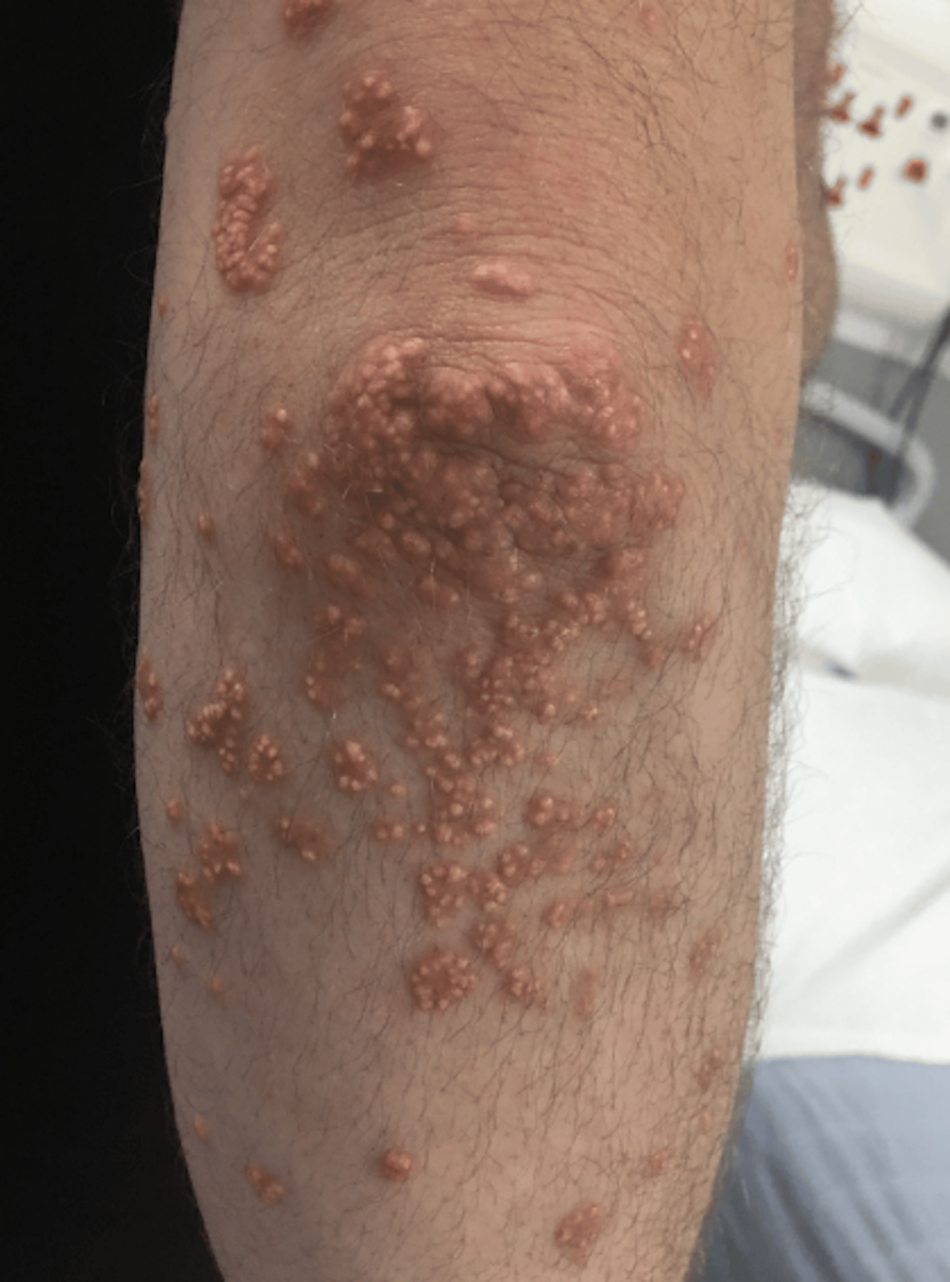
Diffuse papular eruptive xanthomas over the elbow at presentation.

**Figure 5:**
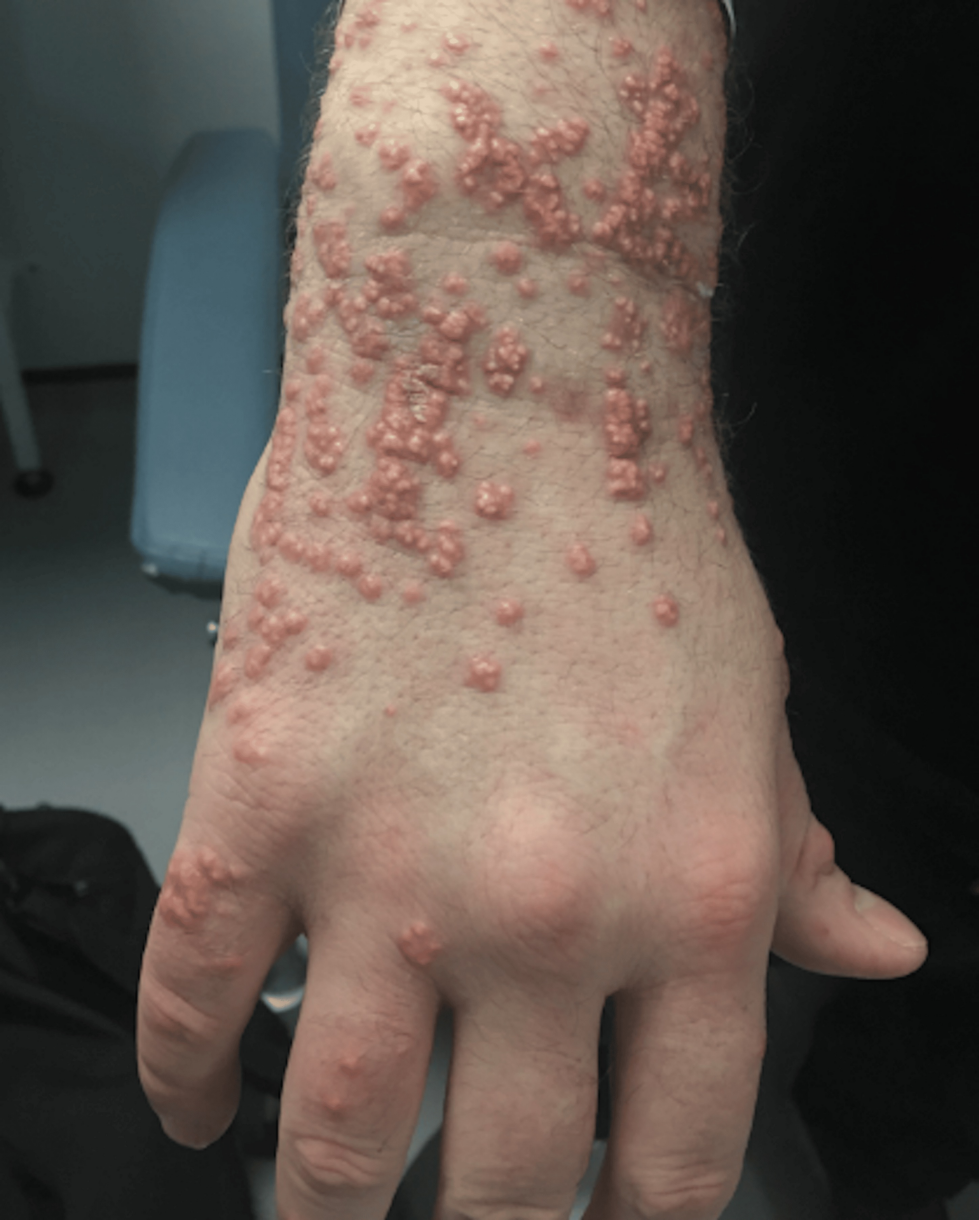
Diffuse papular eruptive xanthomas over the hand at presentation.

On examination, he was hemodynamically stable. Dermatological assessment revealed diffuse papular lesions over the trunk and bilateral upper limbs, consistent with eruptive xanthomas (Figures 1-5).

The patient remained clinically stable, reporting no abdominal pain, nausea, or vomiting, and had no abdominal tenderness on examination. His weight was 113 kg, with a height of 1.75 m, corresponding to a body mass index (BMI) of 35.9 kg/m². Capillary blood glucose levels were elevated, ranging from 16 to 20.3 mmol/L, with ketone levels within normal limits. His HbA1c was markedly elevated at 13.8%, and he was diagnosed with diabetes mellitus at presentation.

Although the patient was clinically well, the option of hospital admission was discussed. Following a shared decision-making process that took into account the patient’s preferences, a mutually agreed plan for outpatient management was established. He was closely monitored with follow-up through the M-SDEC, with early involvement of the metabolic medicine and diabetes teams.

A fasting lipid profile was arranged for further evaluation. The patient was commenced on Humulin I (isophane insulin) at a dose of 8 units twice daily and atorvastatin 40 mg once daily. Follow-up was organised via M-SDEC, including review by the diabetes team, and referrals were made to the lipid clinic. Dietary and lifestyle modification advice was provided, and fenofibrate 160 mg once daily was subsequently added to his treatment regimen.

The results of serial investigations during the course of treatment, including triglycerides, total cholesterol, and HbA1c, are shown in Table 3. 

**Table 3:** Trend of triglycerides, total cholesterol, and HbA1c during treatment HbA1c: glycated hemoglobin

Investigations	Day of referral	3 months later	5 months later	8 months later	A year later	Reference range
Triglycerides	60.57 mmol/L	2.85 mmol/L	5.28 mmol/L	7.50 mmol/L	3.97 mmol/L	0-2 mmol/L
Total cholesterol	21.3 mmol/L	3.9 mmol/L	4.3 mmol/L	4.1 mmol/L	2.9 mmol/L	<5 mmol/L
HbA1c	13.2%	9.3%	9.6	9.3	7.2	4.0-5.9%

Following the diagnosis, the patient completely stopped alcohol intake. With treatment, there was significant improvement in the cutaneous manifestations. He was also referred to the diabetic foot clinic due to symptoms suggestive of diabetic neuropathy. Further improvement in the generalised eruptive xanthomas was observed within one month of improved glycemic control.

At follow-up, there was a significant improvement in triglyceride levels, HbA1c, and cholesterol levels. The skin lesions had completely resolved. Notably, he did not develop acute pancreatitis despite markedly elevated triglyceride levels and extensive eruptive xanthomas. He was successfully managed in the outpatient setting.

## Discussion

Xanthomas develop as aggregates of lipid-laden foam cells within the connective tissue of the skin, tendons, and fascia, and may occasionally involve the periosteum. They are frequently linked to inherited or acquired lipid disorders and can be indicative of certain conditions [2]. 

Xanthomas can be classified using metabolic, patho-anatomical, and clinical criteria [2]. Traditionally, they are broadly categorized into hyperlipidemic and normolipidemic types, based on their association with serum lipid levels [8]. Necrobiotic xanthogranuloma (NXG), however, is considered a distinct entity due to its unique clinico-pathological features and lack of a primary association with lipid abnormalities [2,8]. This distinction reflects differences in underlying pathogenesis and clinical presentation. NXGs usually present as diffuse flat skin lesions. Hyperlipidemic xanthomas are more variable in appearance, often tuberous, and may involve the skin, tendons, or joints [2]. 

For clinical purposes, a second classification based on morphological appearance has been proposed. In this scheme, xanthomas are divided into papulonodular and plane forms. Papulonodular xanthomas include xanthoma eruptivum, tuberosum, tendineum, and articulare, while plane xanthomas comprise xanthoma diffusum planum, intertriginosum, striatum palmare, disseminatum, and xanthelasma palpebrarum. This classification is based solely on the macroscopic characteristics of the lesions [2]. 

The different types of xanthomas, together with their clinical features, associated conditions, lipid status, and classification, are summarised in Table 4. 

**Table 4:** Types of xanthomas: clinical features, associated conditions, lipid status, and classification Adapted from: Zak et al., 2014 [2]

Xanthoma Type	Clinical Features	Associated Conditions	Lipid Status	Classification
Xanthoma eruptivum	Sudden eruption of yellowish papules (1–4 mm) with erythematous halo. Predilection: buttocks, posterior thighs, elbows, lumbar region. Appears within 3 weeks of triglyceride rise.	Severe hypertriglyceridemia (TG >11.2 mmol/L); may signal chylomicronemic syndrome.	Severe hypertriglyceridemia	hypertriglyceridemia Hyperlipidemic xanthoma (HX)
Xanthoma tuberosum	Flat or elevated yellowish nodules (3 mm to several cm) in dermis/subcutaneous tissue. Common over joints (elbows, knees, hands, feet) and buttocks.	Autosomal dominant hypercholesterolemia, familial dysbetalipoproteinemia, β-sitosterolemia, cerebrotendinous xanthomatosis; rarely nephrotic syndrome or hypothyreosis.	Associated with dyslipoproteinemias	Hyperlipidemic xanthoma (HX)
Xanthoma tendineum	Hard, movable nodules or spindle-shaped thickenings in tendons, ligaments, fascia, periosteum. Predilection: Achilles tendon, extensor tendons of hands/fingers, elbows, knees, heels; may develop subperiosteally at tibial tuberosity.	Same dyslipidemias as tuberous xanthomas, except familial dysbetalipoproteinemia.	Associated with dyslipidemias	Hyperlipidemic xanthoma (HX)
Xanthoma diffusum planum	Yellow to orange bands or plates in dermis. Common in axillae, neck, shoulders, buttocks. Rare type.	Usually not connected with dyslipidemia; may indicate monoclonal gammopathy or lymphoproliferative disorders.	Usually normolipidemic	Normolipidemic xanthoma (NX)
Xanthoma striatum palmare	Yellow to orange oblong structures in palmar flexion lines or yellowish discoloration of flexion lines.	Almost pathognomonic for primary dysbetalipoproteinemia; may be seen in newly diagnosed diabetes mellitus, hypothyreosis, primary biliary cirrhosis. Also seen in chronic cholestasis (neck xanthomas, multiple xanthelasmas).	Associated with primary dysbetalipoproteinemia	Hyperlipidemic xanthoma (HX)
Xanthoma disseminatum	Chronic benign cutaneous condition (rare histiocytosis). Small orange-yellow, brown-red or blue-violet papules and nodules. Common in face (periorbital, perioral) and intertriginous areas. May have extracutaneous involvement (CNS, hypophysis, respiratory airways).	Rare histiocytosis syndrome; preferentially affects males in childhood/adolescence.	Not described as associated with dyslipidemia	Normolipidemic xanthoma (NX)
Xanthelasma palpebrarum	Small yellowish, flat or minimally elevated plaques on upper (70%) or lower eyelids. May become circular lesions if spreading. Most common cutaneous xanthoma.	In children/young adolescents: may indicate autosomal dominant hypercholesterolemia (with arcus lipoides corneae, tuberous and tendinous xanthomas). In adults >50: ~50% have dyslipidemia (high LDL-C, high TG, low HDL-C, low apo A-1).	Often associated with dyslipidemia; may occur without it	Plane xanthoma; may be normolipidemic or hyperlipidemic
Necrobiotic xanthogranuloma (Xanthogranuloma necrobioticum)	Progressive granulomatous disorder. Multiple orange-yellow, brownish-red or blue-violet plaques and nodules. Predilection: peri-orbital region; may affect head, neck, trunk. Tendency to ulceration. Histology: Touton giant cells and necrotic foci.	Usually associated with normolipidemia; may signal monoclonal gammopathy or lymphoproliferative malignancies.	Usually normolipidemic	Necrobiotic xanthogranuloma (NXG)

The diagnosis of xanthomas is generally straightforward and is primarily based on clinical findings. In uncertain cases, a skin biopsy with histopathological examination may be required [2]. In this case, the patient presented with eruptive xanthomas, a form that typically appears as sudden clusters of yellowish papules one to four millimetres in diameter surrounded by an erythematous halo. Common sites are the buttocks, posterior thighs, elbows, and the lumbar region [2]. In the present patient, the eruption of multiple small papules in the typical sites supports the diagnosis of eruptive xanthomas. 

The pathogenesis is thought to result from hypertriglyceridemia and chylomicronemia. Primary forms include familial chylomicronemia, corresponding to Fredrickson type I, and primary mixed hyperlipidemia, corresponding to type V, often due to lipoprotein lipase deficiency. Secondary acquired hypertriglyceridemia is commonly related to obesity, heavy alcohol use, and poorly controlled type 2 diabetes [3]. Eruptive xanthomas may also occur secondary to systemic diseases such as diabetes mellitus [6]. In this case, the patient was obese and was newly diagnosed with diabetes mellitus at presentation, evidenced by a markedly elevated HbA1c and raised random blood glucose levels. He also reported increased alcohol intake.

Prevention of xanthomas is achieved through appropriate management of the underlying lipid metabolism disorder. According to evidence-based medicine principles, statins are the first-line therapy in individuals with significant lipid abnormalities. In cases of severe hypercholesterolemia, combination therapy with a statin together with ezetimibe and/or a bile acid resin is recommended. In patients with severe hypertriglyceridemia, fibrates or niacin reduce the risk of acute pancreatitis [2]. High intake of omega-3 fatty acids represents an alternative therapeutic option for severe hypertriglyceridemia [9]. 

Statins are the first-line pharmacological agents for both primary and secondary prevention of cardiovascular disease. They are potent low-density lipoprotein (LDL)-lowering medications that inhibit hepatic cholesterol synthesis and upregulate LDL receptors, thereby enhancing the clearance of LDL from the bloodstream. In addition, statins reduce very low-density lipoprotein levels. Overall, they decrease LDL cholesterol concentrations by approximately 25-55% [9]. Fibrates are mainly used to lower triglyceride levels and reduce very-low-density lipoprotein cholesterol. Clinical experience supports their use in managing severe hypertriglyceridemia to prevent acute pancreatitis. Combining a statin with a fibrate is an effective strategy for treating mixed hyperlipidemia, as it produces a beneficial effect on the overall lipoprotein profile [9]. 

Untreated diabetes mellitus may lead to hypertriglyceridemia through several mechanisms. Insulin deficiency and glucagon excess are associated with impaired lipoprotein lipase activity, resulting in reduced clearance of triglyceride-rich lipoproteins and subsequent hypertriglyceridemia. Severe insulin deficiency can also reduce adipose tissue lipase activity, further contributing to elevated triglyceride levels. Insulin therapy has been shown to reduce triglyceride levels by approximately 50% through restoration of lipoprotein lipase activity and improvement in triglyceride metabolism [10]. Recent studies have demonstrated the efficacy of novel therapies such as olezarsen in reducing triglyceride levels and the risk of acute pancreatitis in patients with severe hypertriglyceridemia [11]. However, this treatment was not utilized in the current case.

The patient’s triglyceride levels were markedly elevated, raising concern for acute pancreatitis. Fenofibrate was added to statin therapy, which led to a significant reduction in triglyceride levels and improvement in the eruptive xanthomas. Although severe hypertriglyceridemia in patients with diabetes may prompt urgent hospital admission because of the theoretical risk of pancreatitis, this patient was successfully managed as an outpatient with close follow-up in the lipid clinic. Management focused primarily on improving glycemic control, which also helped to lower the triglyceride levels. Importantly, he did not develop acute pancreatitis. 

## Conclusions

Eruptive xanthomas are an important clinical marker of severe underlying metabolic disturbance, particularly uncontrolled diabetes mellitus and hypertriglyceridemia. This case highlights that, in the absence of systemic complications such as acute pancreatitis, even markedly elevated triglyceride levels can be safely and effectively managed in an outpatient setting with close monitoring. The rapid improvement in both biochemical parameters and cutaneous manifestations following optimisation of glycemic control, initiation of lipid-lowering therapy, and lifestyle modification, particularly alcohol cessation, underscores the importance of addressing secondary causes of hypertriglyceridemia. 

Importantly, this case highlights that, in carefully selected patients who are clinically stable, an individualized, risk-based approach to management may be considered. In this instance, outpatient management was undertaken following shared decision-making, taking into account the patient’s preferences, clinical stability, and the availability of close monitoring with early follow-up and specialist input. While hospital admission remains standard practice, this case suggests that alternative approaches may be feasible in select circumstances

## References

[1] Martínez DP, Díaz JO, Bobes CM (2008). Eruptive xanthomas and acute pancreatitis in a patient with hypertriglyceridemia. Int Arch Med.

[2] Zak A, Zeman M, Slaby A, Vecka M (2014). Xanthomas: clinical and pathophysiological relations. Biomed Pap Med Fac Univ Palacky Olomouc Czech Repub.

[3] Seremet S, Gurel MS (2018). Miscellaneous skin disease and the metabolic syndrome. Clin Dermatol.

[4] Leaf DA (2008). Chylomicronemia and the chylomicronemia syndrome: a practical approach to management. Am J Med.

[5] Sandhu S, Al-Sarraf A, Taraboanta C, Frohlich J, Francis GA (2011). Incidence of pancreatitis, secondary causes, and treatment of patients referred to a specialty lipid clinic with severe hypertriglyceridemia: a retrospective cohort study. Lipids Health Dis.

[6] Kuo CC, Tsai CW, Su TC (2011). Diabetic eruptive xanthoma. Acta Clin Belg.

[7] Oh RC, Lanier JB (2007). Management of hypertriglyceridemia. Am Fam Physician.

[8] Szalat R, Arnulf B, Karlin L (2011). Pathogenesis and treatment of xanthomatosis associated with monoclonal gammopathy. Blood.

[9] (2014). An International Atherosclerosis Society position paper: global recommendations for the management of dyslipidemia--full report. J Clin Lipidol.

[10] Smith SR (1981). Severe hypertriglyceridaemia responding to insulin and nicotinic acid therapy. Postgrad Med J.

[11] Stroes ES, Alexander VJ, Karwatowska-Prokopczuk E (2024). Olezarsen, acute pancreatitis, and familial chylomicronemia syndrome. N Engl J Med.

